# Untangling unexpected terrestrial conservation challenges arising from the historical human exploitation of marine mammals in the Atlantic sector of the Southern Ocean

**DOI:** 10.1007/s13280-022-01782-4

**Published:** 2022-09-01

**Authors:** Peter Convey, Kevin A. Hughes

**Affiliations:** 1 British Antarctic Survey, NERC, High Cross, Madingley Road, Cambridge, CB3 0ET UK; 2 Department of Zoology, University of Johannesburg, Auckland Park 2006, South Africa

**Keywords:** Antarctic fur seal, Climate change, Conservation, Environmental protection and management, Human impacts, Terrestrial ecosystems

## Abstract

Intensive human exploitation of the Antarctic fur seal (*Arctocephalus gazella*) in its primary population centre on sub-Antarctic South Georgia, as well as on other sub-Antarctic islands and parts of the South Shetland Islands, in the eighteenth and nineteenth centuries rapidly brought populations to the brink of extinction. The species has now recovered throughout its original distribution. Non-breeding and yearling seals, almost entirely males, from the South Georgia population now disperse in the summer months far more widely and in higher numbers than there is evidence for taking place in the pre-exploitation era. Large numbers now haul out in coastal terrestrial habitats in the South Orkney Islands and also along the north-east and west coast of the Antarctic Peninsula to at least Marguerite Bay. In these previously less- or non-visited areas, the seals cause levels of damage likely never to have been experienced previously to fragile terrestrial habitats through trampling and over-fertilisation, as well as eutrophication of sensitive freshwater ecosystems. This increased area of summer impact is likely to have further synergies with aspects of regional climate change, including reduction in extent and duration of sea ice permitting seals access farther south, and changes in krill abundance and distribution. The extent and conservation value of terrestrial habitats and biodiversity now threatened by fur seal distribution expansion, and the multiple anthropogenic factors acting in synergy both historically and to the present day, present a new and as yet unaddressed challenge to the agencies charged with ensuring the protection and conservation of Antarctica’s unique ecosystems.

## Introduction

Antarctica hosts a complexity and antiquity of terrestrial biodiversity and biogeography that has remained widely unappreciated until the last decade. Terauds et al. (2012) and Terauds and Lee (2016) defined 16 distinct terrestrial Antarctic Conservation Biogeographic Regions (ACBRs) within the area of Antarctic Treaty governance. In parallel with this strong regionalisation of Antarctic biogeography, it is also now appreciated that much of the terrestrial biodiversity contained within the ACBRs is endemic at regional and even sub-regional scales, with ancient evolutionary origins and divergences within the continent (Convey et al. 2020). These regions, and their contained biodiversity, are therefore of high conservation value, and the ACBRs are recognised as key conservation tools by the Committee for Environmental Protection (CEP) of the Antarctic Treaty Consultative Meetings, the governance body of Antarctica.

Of the 16 ACBRs, five are located in the western coastal regions of the Antarctic Peninsula and the Scotia Arc, a region more generally known as the maritime Antarctic. The region’s terrestrial ecosystems, which are present on the ~ 1.4% of its area that is seasonally free of snow and ice, are well developed and are characterised by biological components (cryptogamic groups—bryophytes, lichens, algae—biological [cyanobacterial and microbial] soil crusts and small invertebrates) that are fragile and sensitive to both physical damage and pollution. The dominant vegetation comprises bryophytes (mosses and liverworts), which lack the roots of higher plants and are only loosely connected to the underlying substrate. This vegetation and the soil crusts are vulnerable to being damaged or dislodged, while the apparently more robust lichens, many of which are firmly attached to rock and stone surfaces, are also vulnerable to physical fragmentation, particularly when trampled in their commonly desiccated state. The two flowering plants native to Antarctica, the grass *Deschampsia antarctica* and cushion plant *Colobanthus quitensis*, have greater resilience to physical disturbance (in particular, *D. antarctica*) though both can still suffer from crushing, fragmentation and manuring.

In recent decades, vulnerable coastal Antarctic terrestrial and lacustrine communities have been severely damaged by the trampling impacts of rapidly increasing fur seal numbers in the region. In this paper, we (i) describe the damage caused by fur seals to terrestrial ecosystems, (ii) unpick the sequence of human activities that led to the increase in fur seal numbers and distribution, (iii) detail earlier policy and management interventions and (iv) briefly discuss the future challenge of protecting Antarctic terrestrial habitats, recognising the need to use caution when considering human intervention in complex ecological systems.

## Fur seal and human impacts on Antarctic terrestrial ecosystems

**Fig. 1:**
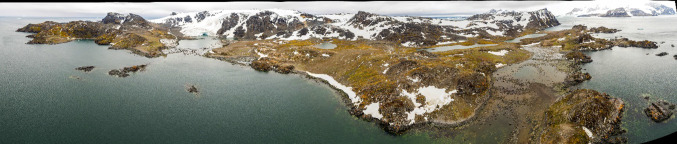
Panoramic view of Signy Island (South Orkney Islands) from the east. With the exception of Observation Bluff (at the far left of the picture), previously extensive vegetation across virtually all of the low lying and accessible ground in the mid- and foreground rapidly suffered heavy damage (estimated as > 75% complete loss or severe damage by Smith (1988a, b)) from fur seal trampling and manuring, which commenced in the late 1970s. Photomontage prepared by A.P. Taylor and S. Adlard

**Fig. 2:**
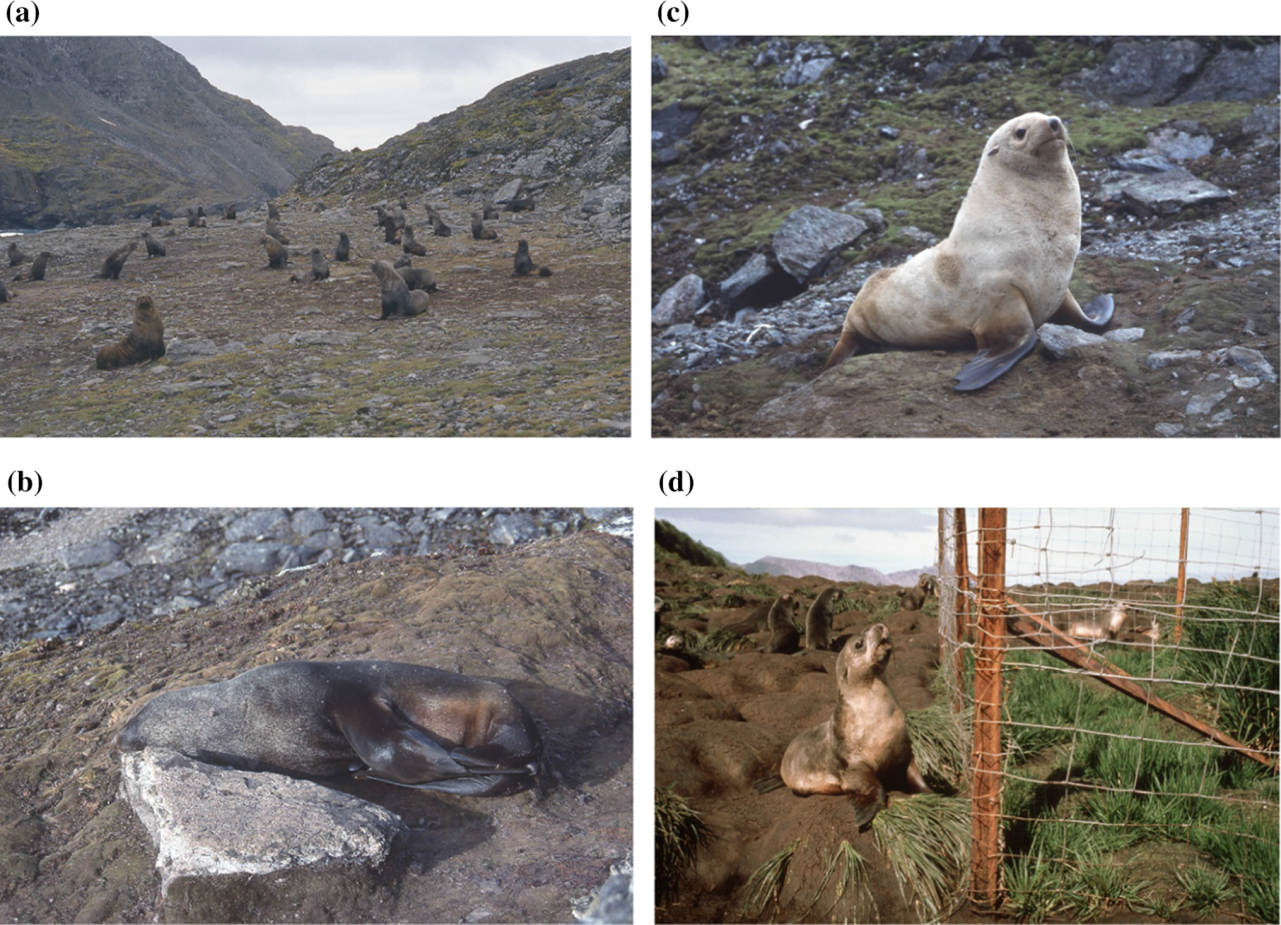
Illustrations of the form of damage caused by Antarctic fur seals to regional terrestrial ecosystems. **a** Hauling out of a dense group of male seals leads to extensive trampling/crushing and fragmentation, as well as over-fertilisation from faeces and urine (brown colouration), of an accessible coastal terrace on Lynch Island, South Orkney Islands (photo: P. Convey, February 1990); **b**, **c** individual fur seals preferentially select even small areas of vegetated ground as resting sites, rapidly crushing and destroying the existing vegetation (photos: P. Convey, January 1991); **d** the boundary of a seal exclosure on Bird Island, South Georgia, illustrating the direct impact of trampling by the recovered fur seal population on native tussac grass vegetation that was able to develop in more accessible coastal locations following the near extinction of the seals in the exploitation era (photo: British Antarctic Survey)

**Fig. 3:**
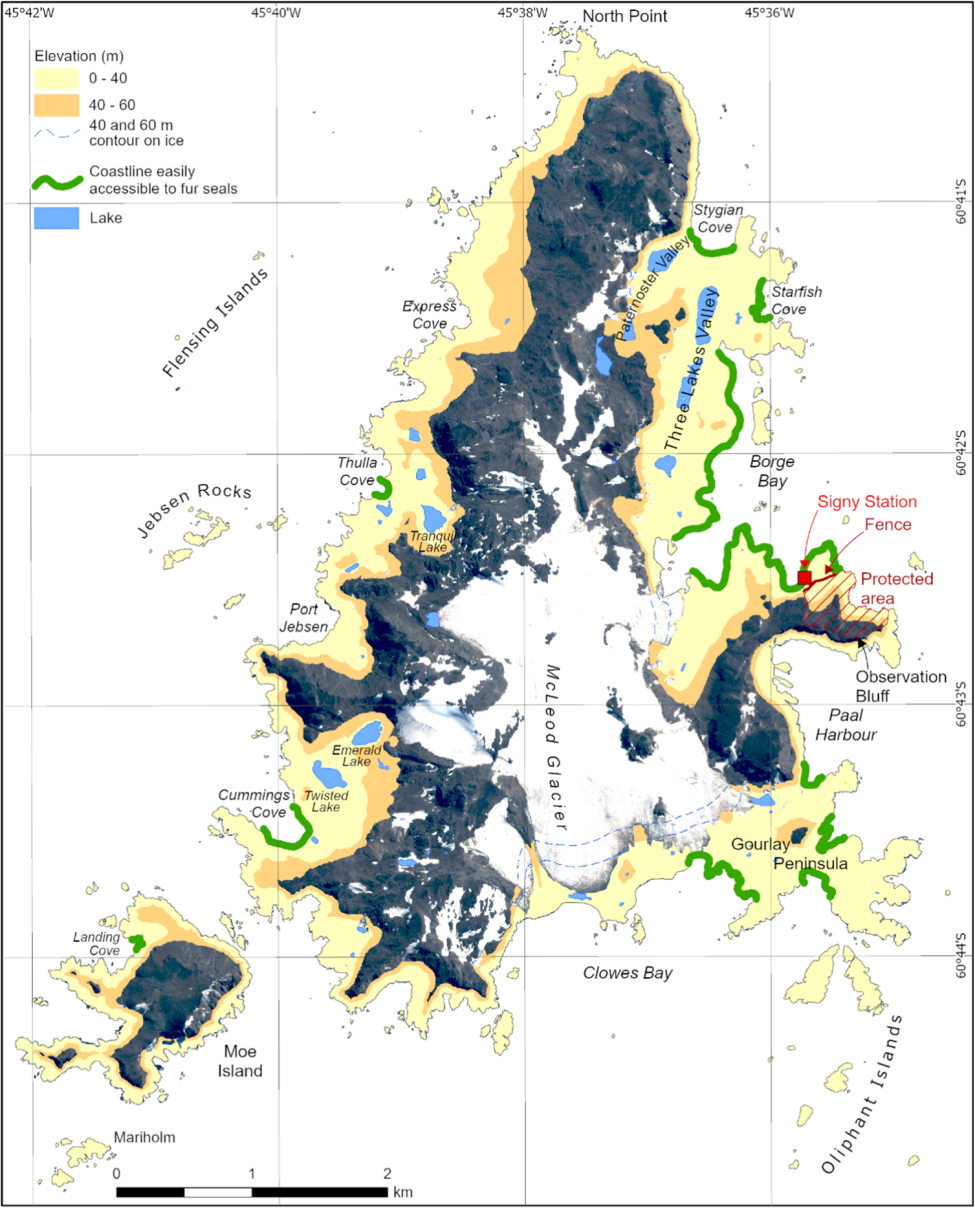
Map of Signy Island indicating the extent of low altitude areas easily accessible from the coast to fur seals. Following Cannone et al.’s (2016, 2017) findings that most vegetation damage occurs below 40 m a.s.l., but is apparent up to 60 m a.s.l., areas within these altitudinal ranges are indicated by yellow and orange shading, highlighting the eastern lowland areas of the island shown in Fig. 1, along with Cummings Cove in the south-west, that have suffered most impacts. Infrastructure on the island currently consists of the summer-operating research station, which typically hosts 5–8 staff for four months each summer. The island is accessed on foot from the station, with primary marine bird research and monitoring sites around the Gourlay Peninsula and North Point. The area actively protected from seal impact by construction of a fence on Berntsen Point is also indicated by red diagonal shading

While research has been undertaken on the impacts of human activities on Antarctic vegetation and soils (Tin et al. 2009; Tejedo et al. 2012), less is known about those of the Antarctic fur seal (*Arctocephalus gazella*) whose presence has recently and rapidly expanded in the region since the mid-1970s. One location where fur seal impacts have been observed and studied in some detail for several decades is Signy Island in the South Orkney Islands. Here, changes in lichen diversity have occurred as a result of the substantial increase in trampling by fur seals transiently occupying the island each summer; at local scale, these include both reduced abundance and losses of species sensitive to physical disturbance and/or excessive nutrient contamination and increases in species positively associated with nutrient enhancement (Favero-Longo et al. 2011). Considerable damage to and loss of previously extensive cryptogamic vegetation and also grass on large areas of relatively flat ground at lower altitudes easily accessible from the coast have also been documented on the island and in the neighbouring Antarctic Specially Protected Area (No. 110) of Lynch Island (Smith 1988a, 1990, 1996a, 1996b, 1997; Cannone et al. 2016, 2017) (Figs. 1, 2, 3) as well as at other locations along the Antarctic Peninsula (Smith 1996a, b).

Most Antarctic terrestrial ecosystems are characterised by being highly nutrient-limited (Convey et al. 2014), while lakes are generally oligotrophic, with some being ultra-oligotrophic (Heywood 1967; Heywood et al. 1980; Butler 2000; Quayle and Convey 2006; Izaguirre et al. 2021). Wind-blown fertilisation by marine-derived nutrients can occur over distances of several hundred metres from vertebrate concentrations, such as penguin colonies and seal wallows, and has been shown to be a positive driver of Antarctic terrestrial biodiversity (Bokhorst et al. 2019). However, if this fertilisation is accompanied by the intense trampling and excessive manuring experienced within and very close to colonies or wallows, most terrestrial diversity is rapidly eradicated at this local scale, often being replaced by the terrestrial foliose alga *Prasiola crispa*. Similarly, the recent presence of large numbers and densities of fur seals on islands such as Signy Island has led to rapid eutrophication of more accessible lakes close to the coast, which the seals utilise (Ellis-Evans 1990; Hawes 1990; Butler 1999; Quayle and Convey 2006).

The fragility of these terrestrial ecosystems is a recognised conservation issue and challenge in Antarctica. This challenge is driven by the combination of multiple factors, including the small overall amount of ice-free ground available, the small proportion of that area that hosts vegetation development, the nature of that vegetation and the competition resulting from the increasing demand for suitable ice-free locations where human activity is concentrated (Tin et al. 2009; Hughes et al. 2016; Brookes et al. 2019). It has been estimated that as little as 1.34% of ice-free ground on the western Antarctic Peninsula is vegetated (Fretwell et al. 2011). This estimate does not include the South Shetland Islands and South Orkney Islands, which host some of the richest terrestrial communities in the maritime Antarctic (Smith 1988a, 1990; Øvstedal and Smith 2001; Ochyra et al. 2008). Furthermore, Hughes et al. (2016) highlight that formal protection of vegetated ecosystems within the Antarctic Specially Protected Area (ASPA) system is both extremely limited and very uneven, with a total of only c. 16 km^2^ of vegetation protected within ASPAs across the entire continent, of which half is contributed by a single ASPA (No. 126 Byers Peninsula, Livingston Island). The Antarctic Treaty System’s Committee for Environmental Protection (CEP) and the independent Scientific Committee on Antarctic Research (SCAR) have developed guidelines that clearly recognise the vulnerability of these ecosystems and the need for careful management and avoidance of human impact on them (SCAR 2018). Nevertheless, many instances of such human damage, which can remain apparent for decades, have been and continue to be reported both beyond and within ASPAs, with many more not formally recorded (Smith et al. 1994; Tin et al. 2009; Braun et al. 2012, 2014; Peter et al. 2013; Convey 2020; Finger et al. 2021).

The ‘footprint’ of human activities is particularly significant and concerning in the South Shetland Islands and north-west Antarctic Peninsula, where multiple nations operate logistic hubs, research stations and field facilities, and tourism operators use regular visitor sites. For instance, virtually all ice-free areas or significant headlands in the South Shetland archipelago host research stations, refuges, camp sites, field instrumentation, regular research sites, or have become well-established visitor sites. Several ASPAs in the South Shetland Islands include semi-permanent field camps, refuges or field sites of national operators which are routinely used in support of scientific research (e.g. ASPAs 126 Byers Peninsula, 112 Coppermine Peninsula, 133 Nelson Island, 151 Lions Rump) or are immediately adjacent to research stations and/or some of the most visited tourist sites (e.g. 140 Deception Island, 150 Ardley Island, 128 Western shore of Admiralty Bay). All of this activity leads to human impacts of varying intensity, including within protected areas.

The major elements of anthropogenic impact and environmental change facing Antarctica, in particular the Antarctic Peninsula, and its ecosystems are arguably well recognised (Lee et al. 2017; Convey and Peck 2019; Siegert et al. 2019). However, frustration continues to be expressed regarding the slow pace of response within the Antarctic Treaty System in a number of areas relevant to environmental protection. Examples include the development of a systematic, effective and representative protected area system (Shaw et al. 2014; Hughes et al. 2016; Coetzee et al. 2017) and the prevention of further and mitigating existing anthropogenic damage to ecosystems, especially those in the vicinity of concentrations of human activity (Peter et al. 2008, 2013; Tin et al. 2009; Hughes and Convey 2014; Convey 2020). Other challenging issues include controlling the further expansion of human influence and cumulative impact on the Antarctic environment, with the inexorable expansion of research and tourism activities to ever more remote parts of the continent (Hughes et al. 2011; Pertierra et al. 2017; Brooks et al. 2019; Leihy et al. 2020) and the expansion of existing, and construction of entirely new, research stations and logistic facilities, which have historically faced little limitation in practice. The aforementioned activities have largely commenced despite the Protocol on Environmental Protection’s mandated protocols of assessment of environmental impacts and consultation of Treaty Parties (Lyons 2009; Hemmings and Kriwoken 2010), in part as it is also the case that the outcomes of the consultation process are only advisory and the Treaty has no enforcement powers.

## Southern Ocean marine resource exploitation

**Fig. 4:**
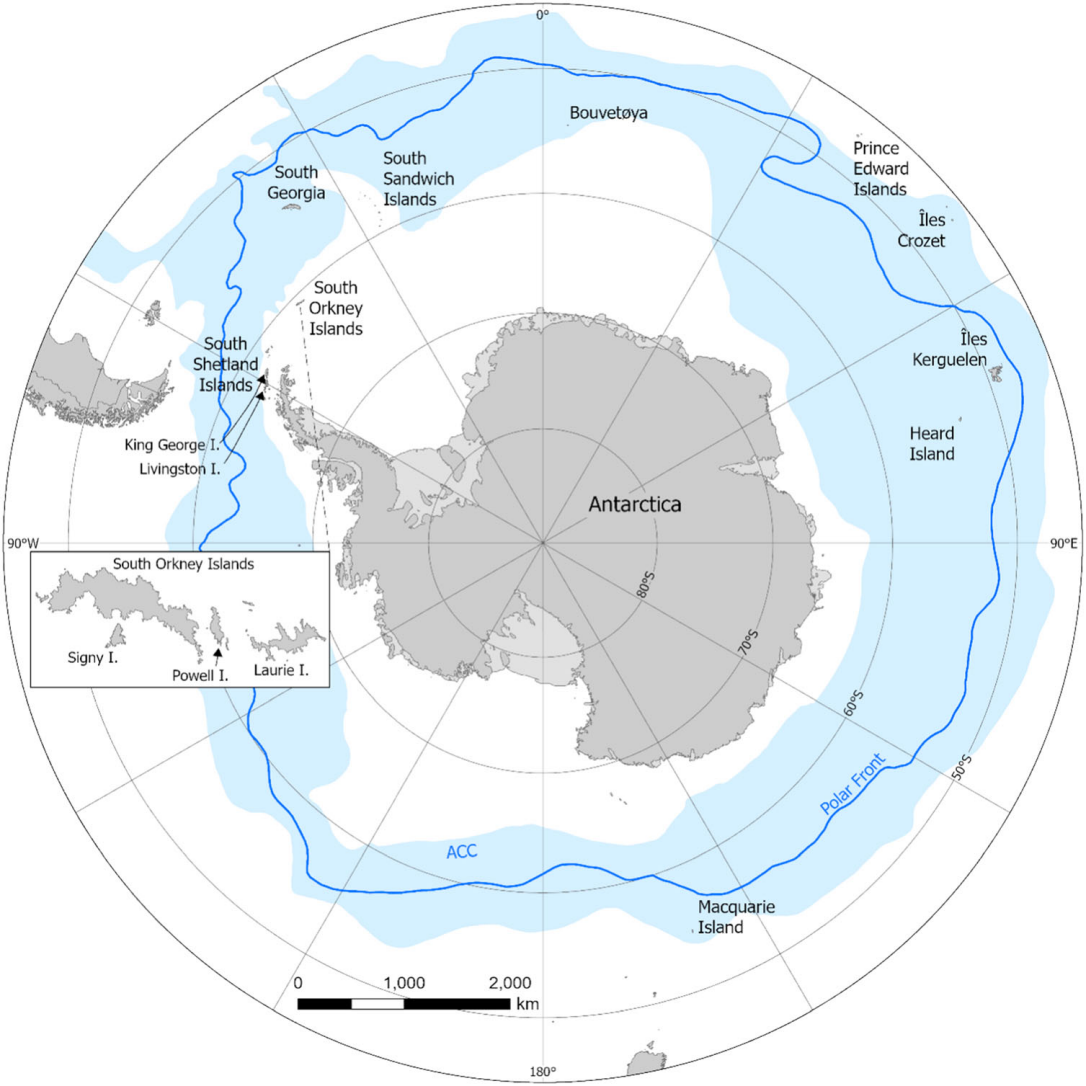
Map of the Southern Ocean around Antarctica showing the islands and island groups hosting breeding populations of Antarctic fur seals that were exploited around the northern Antarctic Peninsula, the Scotia Arc archipelagos and the sub-Antarctic islands. The primary population centre is South Georgia, hosting more than 95% of the global population of the species

Large-scale human impacts in the Southern Ocean regions commenced within a few years of the discovery of the remote sub-Antarctic islands in the latter part of the eighteenth century, quickly followed by the South Shetland Islands in the early nineteenth century. These discoveries took place in a very different era to the present day, driven by imperialism, the search for new territory and for opportunities to exploit new sources of valuable resources. The Southern Ocean rapidly became a primary target of this exploitation throughout the nineteenth century, driven in particular by increasing demand for the pelts of fur seal species found in the Antarctic and sub-Antarctic (*Arctocephalus gazella*, *A. tropicalis*) and oil from elephant seals (*Mirounga leonina*), high value commodities at that time (Bertrand 1971; Headland 1984, 2018; Townrow 1988; Trathan and Reid 2009). Large populations of fur seals on the sub- and other peri-Antarctic islands, but particularly on South Georgia and the South Shetland Islands (Fig. 4), were the first to suffer uncontrolled overexploitation, almost being driven to extinction (Bonner 1968; Forcada and Staniland 2009; Paijmans et al. 2021; Krause et al. 2022). The sequence of rampant overexploitation was followed by the great whales, with the development of, initially, shore-based whaling stations on several peri-Antarctic islands in the early twentieth century, and later of the pelagic whaling industry (e.g. Basberg 2004; Hart 2006). The exploitation of elephant seals (*Mirounga leonina*) was generally less intense and ultimately led to one of the first examples of pre-emptive management (Laws 1994). After the destruction of whale populations and demise of the whaling industry by the mid-1960s, overexploitation of marine resources continued through fisheries, with populations of some fish species being reduced to the extent that even in 2022, 40 + years after the establishment of the Commission for the Conservation of Antarctic Marine Living Resources (CCAMLR) in 1982, full recovery has not yet occurred (Agnew and Nichols 1996; Grant et al. 2021).

## Wider ecosystem impacts of marine overexploitation

One key consequence of this history of catastrophic overexploitation was its fundamental impact on the entire Southern Ocean marine ecosystem to the extent that, even now, its pre-exploitation structure and functioning remain unclear (Grant et al. 2021). Furthermore, it is not clear what trajectory ecosystem recovery will ultimately follow, even before the potential impacts of contemporary physical environmental change are considered (Griffiths et al. 2017; Convey and Peck 2019; Siegert et al. 2019; Morley et al. 2020; Chown et al. 2022; but see also Cavanagh et al. 2021). However, after large-scale disturbance, ecosystem theory suggests that recovery may lead to alternative stable states rather than simply a return to the original state (May et al. 1979; Bender et al. 1984; Mori 2011; Henderson et al. 2016; Yi and Jackson 2021).

The ramifications of this previous major human impact spread much farther beyond the Southern Ocean than is generally appreciated, with key impacts to the present day on other, non-marine, Antarctic ecosystems, which pose currently largely unaddressed conservation and management challenges. In their sub-Antarctic breeding and hunting grounds, both seal and whale exploitation had potentially considerable, but entirely unknown and undocumented, impacts on local terrestrial ecosystems at the time (see Convey and Lebouvier (2009) for discussion). With the size of pre-exploitation fur seal populations unknown, but thought to be of similar magnitude to those now existing after recovery (Forcada and Staniland 2009; Foley and Lynch 2020), and in the absence of significant native terrestrial vertebrates (Headland 1984; Convey 2017), the near extinction of fur seals would have greatly reduced their trampling and fertilisation of accessible terrestrial ecosystems. Aside from the concentrated impacts within dense breeding colonies, non- and post-breeding seals haul out in neighbouring coastal terrestrial habitats and can impact extensive areas hundreds of metres from the coastline, as well as to at least 60 m altitude, as illustrated in Fig. 2d (see Bonner 1985; Cannone et al. 2016). Such coastal areas host the highest development in terms of biomass and biodiversity of most Antarctic and sub-Antarctic native terrestrial vegetation and invertebrate communities. In South Georgia, where Antarctic fur seal populations have recovered and now represent more than 95% of the global population (Forcada and Staniland 2009), they have shown rapid expansion in the local occupied range over the last two to three decades around the coasts of the island (Payne 1977; see also discussion in Trathan et al. 2012) and this form of trampling damage is increasingly apparent both on South Georgia and at more southern locations (Smith 1988a, 1997; Favero-Longo et al. 2011; Cannone et al. 2016, 2017).

However, observations of recent trampling increase in the seals’ pre-exploitation breeding range on South Georgia also highlight that the contemporary perception of ‘typical’ sub-Antarctic coastal terrestrial habitats on this island may be inaccurate, with these now-impacted areas of habitat developing after the end of seal exploitation and persisting through most of the nineteenth and twentieth centuries. While detailed records of seal distribution on South Georgia are not available from this period, an assumption that the distribution of archaeological sealing relics (huts, caves, artifacts, etc.) reflects that of the seals being hunted is plausible. If so, then pre-exploitation seal distributions occupied much of the coastline of South Georgia and also parts of the South Shetland Islands (Headland 2009, 2014, 2018; Senatore 2019), although more physical evidence remains from elephant than fur sealing.

There must also have been major changes in transfer of marine-derived nutrients to land. This would have included short-term increases at a localised scale through the originally common practice of simply dumping carcass remains of both seals and whales on the shoreline where they were scavenged in part by birds such as giant petrels, skuas and sheathbills, thereby transferring nutrients to land in their guano. In the longer-term, decreases in nutrient transfer would also have taken place through the reduction of input of seal- and sometimes penguin-sourced faeces as their colonies, which are intense sources of aerosol dispersal of nutrients (cf. Bokhorst et al. 2019), were wiped out. Marine-derived nutrients are a major driver of terrestrial biodiversity in the sub-Antarctic and Antarctica (Smith [Bibr CR120][Bibr CR125]; Zwolicki et al. 2015; Bokhorst et al. 2019). Thus, such changes in nutrient transfer must have had considerable, but completely undocumented, local impacts on terrestrial biodiversity.

## The specific case of Antarctic fur seal population recovery

As noted, the South Georgian Antarctic fur seal population is thought to have recovered to a level comparable with or perhaps greater than its pre-exploitation level (e.g. Boyd 1993; Forcada and Staniland 2009; Foley and Lynch 2020). The much smaller South Shetland Islands population, the species’ southern-most breeding population, most likely recovered from a very small remnant after near extirpation, possibly reinforced by some migration from South Georgia (Reid et al. 2006; Krause et al. 2022). Recent detailed genetic studies support the two regional populations being distinct (Paijmans et al. 2021). Although the South Shetland Islands population appeared to have stabilised at a level of about one eighth of its pre-exploitation level by the early twenty-first century (Hucke-Gaete et al. 2004; Hoffman et al. 2018), it has subsequently shown a further substantial decrease in numbers suggested to be a consequence of a sustained increase in top-down predation of pups by leopard seals (Krause et al. 2022). However, a study of seal hair abundance in a core of accumulated terrestrial sediment on a coastal raised beach on King George Island (Sun et al. 2004) inferred large levels of variation in the size of the local fur seal population over the last 1500 years. The authors go on to speculate that this variation might relate to climatic factors and suggest that this population was subject to wide natural fluctuations even before the large-scale human destabilisation of the Southern Ocean ecosystem. The even smaller population on the remote and less accessible South Sandwich Islands received less impact from sealers and appeared to have only increased slightly between the only published surveys in the early 1960s and late 1990s (Holdgate and Baker 1979; Convey et al. 1999).

The overall recovery of fur seal populations has possibly been facilitated by the seals reaching sexual maturity much more rapidly than do baleen whales. The two overlap in their primary food source of krill, resulting in there potentially having been a ‘krill surplus’ available to support higher seal and penguin populations that was not available prior to whale overexploitation, although this has not been proven (Croxall 1992; Boyd et al. 1995; Trathan et al. 2012). With increasing evidence that at least some Antarctic whale populations are now recovering (Zerbini et al. 2019; McCormack et al. 2021), it is not clear whether the fur seal population ‘rebound’ will remain at its current level, or whether there will be some form of rebalancing with the increased number of whales. However, the consistent and large recent decreases in fur seal pup numbers and, by implication, adult populations on the South Shetland Islands (Krause et al. 2022) and South Georgia (Forcada and Hoffman 2014) over the last two decades, while having a number of possible contributing causes, may suggest this is happening already. Further important unknowns are the distribution and future trajectory of human exploitation of the krill resource, the primary food source exploited by fur seals and often described as one of the greatest remaining largely unexploited sources of protein on the planet (McBride et al. 2014; Trathan et al. 2021). Krill abundance and distribution are also intimately linked with the impacts of anthropogenic climate change on sea ice extent and distribution (Constable et al. 2014; Atkinson et al. 2019).

## Interaction between fur seals and maritime Antarctic terrestrial ecosystems

An important feature of the Antarctic fur seal’s recovery is that their impact on terrestrial ecosystems now appears to extend to cover a much wider area of the maritime Antarctic, and involves considerably larger numbers of seals, than there is any evidence for having been the case pre-exploitation (Hodgson et al. 1998). There is no evidence that fur seals have occupied these areas and caused such damage previously, certainly since the end of the Pleistocene. The breeding range of this seal species has not changed, centred on South Georgia with far smaller outlying populations on the South Sandwich Islands, Bouvetøya and in parts of the South Shetland Islands as well as on other eastern sub-Antarctic islands (Table 1; Paijmans et al. 2021). The current impacts result from both immature males and post-breeding season bulls, but only very few females, from the South Georgia population dispersing widely to forage and haul out on land during the second half of the austral summer (Boyd et al. 1998; Waluda et al. 2010). They now come ashore in large numbers to rest and moult on the South Orkney Islands as well as on the islands off the north-east Antarctic Peninsula (James Ross Island area) and the length of the western Antarctic Peninsula as far south at least as Marguerite Bay (68–69° S). Conversely, tracking studies have shown that pelagic females and subadult and adult males from the South Shetland Islands population move south along the Antarctic Peninsula towards the end of summer and into the winter period (Arthur et al. 2017). Tracking studies of pelagic seals have not included reports or assessments of seals spending time ashore and it remains the case that there are no monitoring programmes regularly recording numbers of fur seals coming ashore at any location along the Antarctic Peninsula. The farthest south Antarctic fur seal records we are aware of are of single mature males observed on ice floes in the Ronne Entrance, south of Alexander Island (~ 73° S) on 12 February 2008 and in Lazarev Bay (69° 22′ S) on 17 February 2008 (Convey, pers. obs).

**Table 1:** The most recently available estimated sizes of Antarctic fur seal (*Arctocephalus gazella*) populations across the maritime Antarctic and sub-Antarctic

Location	Pup number	Total population	Census season	References
Macquarie Island	148		2007/2008	Goldsworthy et al. (2009)
Heard Island	1278		2003/2004	Goldsworthy (pers. comm.), in SCAR-EGS (2008)
McDonald Island	100	300	1979/1980	Johnstone (1982)
Iles Nuageuses (Iles Kerguelen)	1500–1700		2000	Lea (pers. comm.), in SCAR-EGS (2008)
Ile de la Possession (Iles Crozet)	295		2003/2004	Guinet (pers. comm.), in SCAR-EGS (2008)
Marion Island	796^a^	3821	2003/2004	Hofmeyr et al. (2006)
Prince Edward Island	400	2000	2001/2002	Bester et al. (2003)
Nyrøysa (Bouvetøya)	15 523^a^	66 128	2001/2002	Hofmeyr et al. (2005)
South Georgia		4 500 000 –6 200 000^b,c^	1999/2000	Boyd (pers. comm.), in SCAR-EGS (2008)
South Sandwich Is	1750		1997/1998	Convey et al. (1999)
Signy Is., South Orkney Is	0	12 607	2007/2008	British Antarctic Survey (unpublished)
Laurie Is., South Orkney Is		16 610	2004/2005	Carlini et al. (2006)
South Shetland Is	7602		2007/2008	Goebel et al. (2008)
Cape Shirreff, South Shetland Is	860^d^		2019/2020	Krause et al. (2022)
San Telmo Islet	333		2018/2019	Krause and Hinke (2021)

^a^Corrected for pre-count mortality

^b^Estimated from the number of breeding females

^c^Standard deviation = 300 000

^d^Standard deviation = 11

Long-term records of fur seal numbers in Antarctic locations are largely lacking; however, the South Orkney Islands may be the exception, with some degree of human presence and record keeping for much of the past 100 years or more. There are records of a very small number of fur seals being taken in the South Orkney Islands in the early nineteenth century (Marr 1935), but sealing records from the archipelago are very scarce, perhaps reflecting that it was historically more affected by sea ice extending up from the Weddell Sea. If so, fur seals may have been restricted from reaching the archipelago, as it seems unlikely that sealers would not have exploited any commercially viable population. Following the cessation of nineteenth century exploitation of fur seals on South Georgia and the South Shetland Islands, the first records of fur seals in the South Orkney Islands were from Laurie Island in 1936 (Headland 1989) and from Signy Island in 1948 (Laws 1973). The first thorough survey of the South Orkney Islands in 1971 (Laws 1981) recorded a total of 2035 fur seals. As noted by Hodgson et al. (1998), no reference to fur seal presence was made during the whaling period on Signy Island (1907/1908 to 1928/1929), although it seems likely that any seen would have been harvested. Annual numbers on Signy Island increased from a few dozen in the 1960s to a few hundred by the late 1970s, but then very rapidly increased to around 20,000 in the mid-1990s to early 2000s (maximum 21 303 in 1994; Waluda et al. 2010). Comparable increases in numbers were also reported from neighbouring Laurie Island (Vergani and Coria 1989; Carlini et al. 2006), while Yang et al. (2010) in a short terrestrial sediment study dating from the early twentieth century onwards inferred a similar pattern of increase in the local population on King George Island (South Shetland Islands). In the 1964/1965 summer, a small fur seal colony on Powell Island and neighbouring Michelsen Island in the South Orkney Islands contained around 550 adults and 35 pups, with a further smaller colony with pups on Fredriksen Island, and smaller numbers at Meier Point on Coronation Island and Monroe Island off the western point of Coronation Island (Smith pers. comm.). Laws (1981) reported the results of surveys of most of the accessible coastline of the South Orkney Islands that took place in 1971 and 1974. These surveys confirmed the presence of only three small breeding colonies (Gosling Islands, Monroe Island, Michelsen Island), with totals of 61 and 65 pups in the two years, respectively, and recorded only 12 male fur seals on Signy Island. Notably, the Signy Island numbers do not reflect the presence of a breeding population; the first pup was observed on the island in 1977 but, subsequently and even in the years with the highest counts, only a handful of females and even fewer pups have been recorded. Similarly, throughout the 1990s and 2000s, summer-dispersed male fur seals were reported from progressively more southern locations along the Antarctic Peninsula (e.g. Brabant Island, Furse (1986); Rothera Point, Marguerite Bay, Hughes (2003)), where they had not been reported previously in large numbers.

These observations initially led to a conclusion that no significant population of fur seals had been present on Signy Island or elsewhere in the South Orkney Islands since the retreat of ice after the Last Glacial Maximum (Smith 1988a, 1990). However, lake sediment studies on the island have refined this conclusion, confirming the presence of fur seal hairs in sediments dated up to c. 6500 years old (Hodgson et al. 1998; Hodgson and Johnston 1997) although, as with Sun et al.’s (2004) study on King George Island, with considerable variation over time. Importantly, Hodgson et al. (1998) suggested that the seal population size on Signy Island, as indicated by numbers of hairs retrieved throughout the sedimentary records, was consistently much smaller than that present from the 1980s onwards, concluding that this may be a clear indication of human interference in the Southern Ocean ecosystem. Unfortunately, although palaeolimnological studies have analysed sediment cores spanning the Antarctic Peninsula region from the South Shetland Islands, James, Ross Island, Hope Bay and south to Horseshoe Island, no studies appear to have searched for or reported fur seal hairs in these lake sediments. Similarly, seal hairs have not been reported amongst biological material recovered from studies of raised beaches, or from deep moss peat bank cores.

## The role of climate change in southward fur seal distribution and haul-out site availability

There is no definitive explanation as to why the summer-dispersing male seals from South Georgia have expanded their previous range so dramatically. As noted above, a disturbed ecosystem will not automatically return to its original state after the disturbance, but this range expansion coincides with two particularly significant anthropogenically driven environmental changes in this region. First, the well-known Antarctic Peninsula regional air temperature warming in the second half of the twentieth century that was in large part driven by progressive reduction in extent and duration of winter sea ice west of the Peninsula and throughout the Scotia Arc. This meant that marine buffering of air temperatures over land played a much larger role than previously and, while warming took place in all seasons, the strongest trends were in the winter (Smith and Stammerjohn 2001; Stammerjohn et al. 2008; Turner et al. 2009; Eayrs et al. 2021). The retreat of sea ice may also have been accompanied by a parallel southward shift in distribution of Antarctic krill (*Euphausia superba*), the primary prey of fur seals and whose reproduction is linked with winter sea ice (Atkinson et al. 2019). Second, the warming resulted in ice and snow recession on land, both in terms of extent and earlier timing (Cook et al. 2005; Mulvaney et al. 2012), a process predicted to continue over the next century (Lee et al. 2017; Hughes et al. 2021). However, while ice recession will lead to increased area of habitat that can potentially be colonised by terrestrial biota, this habitat will primarily be formed through the expansion of coastal, low altitude areas that are already accessible to the summer-dispersing fur seals. Together, these two facets of regional environmental change give pelagic fur seals ease of movement and foraging farther south in open water and access to new areas of haul out on land. Interestingly, the previously strong Antarctic Peninsula warming trend paused and even reversed in the early years of the twenty-first century, with a series of colder years with increased snow and ice cover (Turner et al. 2009). This may be another factor leading to fur seal numbers on Signy Island subsequently remaining below the maxima reported in the mid-1990s (Waluda et al. 2010), and recent evidence of some recovery in previously heavily trampled areas of the two native flowering plants on the island (Cannone et al. 2022). Suggestively, Sancho et al. (2017) report similarly rapid, in this case negative, responses of South Shetland Islands lichens to this temporary cooler period. However, both studies serve to highlight how rapidly Antarctic terrestrial ecosystems can respond to different environmental drivers.

## Fur seal policy and management

### Early sealing regulation

By the start of the twentieth century, fur seals were considered all but extinct as a result of earlier overexploitation and no on-going sealing activity was commercially viable. The last commercial fur sealing expedition was to South Georgia in 1907, when 170 pelts were taken (Larson 1920). A British administration was established on South Georgia in 1906 and, while fur seal hunting was prohibited, permits for elephant sealing were granted from 1909 to the mid-1960s during which time c. 250 000 were taken (Hofman 2017). However, from the late 1950s, fur seal numbers on South Georgia had started to recover to a level where there was consideration of allowing renewed harvesting (Bonner 1958).

### Convention for the Conservation of Antarctic Seals

In the early 1960s, exploratory research was undertaken to assess the viability of recommencing sealing in Antarctica (Øritsland 1970). Recognising concerns over the vulnerability of Antarctic seal species to commercial overexploitation and to reduce perturbations in the Antarctic marine ecosystem, in 1972, the Antarctic Treaty Consultative Parties developed the Convention for the Conservation of Antarctic Seals (CCAS) which entered into force in 1978 (available at: https://documents.ats.aq/keydocs/vol_1/vol1_13_CCAS_CCAS_e.pdf). CCAS prohibits the taking of Antarctic seals except in specific circumstances and in accordance with a permit. It established annual catch limits for each seal species, with taking of elephant and fur seals prohibited at any time, and established six sealing zones, a sealing season (1 September to the end of February) and three seal reserves. Contracting Parties are also required to provide annual reports on the sex, reproductive condition and age of seals taken. However, by the time CCAS entered into force in 1978, no sealing industry had developed in Antarctica, and the Convention was later largely superseded by the Protocol on Environmental Protection to the Antarctic Treaty (also known as the Environmental Protocol or Madrid Protocol; agreed in 1991, entered into force 1998) which, in effect, prohibited the commercial exploitation of seals (Annex II).

### Specially Protected Species

Following the entry into force of the Antarctic Treaty in 1961, attention was directed towards conservation issues with the approval of the Agreed Measures for the Conservation of Antarctic Fauna and Flora (1964), which allowed for the addition of any native Antarctic species to the list of Specially Protected Species (SPS) following agreement by the Antarctic Treaty Consultative Meetings (ATCM). Through the Agreed Measures, Specially Protected Species (SPS) status was afforded to all species of the genus *Arctocephalus* (fur seal) within the Antarctic Treaty area (although only *A. gazella* is resident in the area), in response to the drastic population reductions resulting from earlier overexploitation. This high level of protection was continued when the Agreed Measures were used as the basis for the drafting of Annex II ‘Conservation of Fauna and Flora’ to the Environmental Protocol, despite the considerable increase in the fur seal population already documented during the intervening period. However, in 1999, the ATCM asked SCAR to provide a recommendation about the appropriateness of continued listing of fur seals as an SPS (Resolution 2, 1999). SCAR concluded that, on the basis of present populations and trends of these populations, fur seals could not be considered threatened or endangered under the IUCN Red List criteria and therefore were no longer in need of special protection (SCAR 2006). As a result, the ATCM removed fur seals from the list of SPS through Measure 4 (2006). The International Union on Nature Conservation defined the Antarctic fur seal as a species of least concern and not threatened in any part of its distribution in 2014 (Hofmeyr 2016). However, recent research has questioned this decision, on the basis that genetic studies have confirmed the existence of at least four genetically distinct sub-populations (South Shetland Islands, South Georgia, Bouvetøya, eastern sub-Antarctic islands) (Paijmans et al. 2021; Krause et al. 2022). As noted earlier, both the South Georgian and South Shetland Islands populations have consistently declined over the last two decades, and the decline in the latter has recently been described as potentially catastrophic, risking loss of an important component of the species’ genetic diversity (Krause and Hinke 2021; Krause et al. 2022).

## Management of fur seal damage to terrestrial ecosystems

Where they are present on land in previously unoccupied areas during the austral summer, fur seals have major and long-term impacts on the fragile terrestrial vegetation, soils and microbial soil crusts that typify the maritime Antarctic (Block et al. 2009), both through direct trampling and excessive nutrient input from manuring (Fig. 2; Smith 1988a, 1997; Favero-Longo et al. 2011; Cannone et al. 2016, 2017). This often leads to complete destruction of the original communities, as documented for some of the previously best vegetated areas in the maritime Antarctic on Signy Island (Smith 1972, 1988a, 1997), although also encouraging the development of some nitrogen- or trampling-tolerant species such as the alga *Prasiola crispa* and ornithocoprophilous lichens (e.g. see Favero-Longo et al. 2011). In the sub-Antarctic, Haussmann et al. (2013) showed that trampling by the fur seal population on Marion Island can facilitate the local establishment of non-native vascular plants, while Frenot et al. (2001) reported that the non-native grass *Poa annua* had formed low grasslands around elephant seal wallows on sub-Antarctic Île de la Possession (Crozet Islands) and Kerguelen Island. Similarly, trampling and grazing by non-native reindeer have been suggested to have facilitated the wide distribution of *P. annua* in coastal valleys where whaling station activity was concentrated along the north-east coast of South Georgia. While the reindeer have recently been eradicated, the recovering fur seal population has now spread along this coast, providing continuation of the trampling activity. Such establishment events may also be possible at seal impacted sites in the maritime Antarctic, particularly considering the increasingly frequent reports of non-native plant introductions in the region (Hughes et al. 2015; Malfasi et al. 2019).

### Fur seal impacts on ASPAs and ASMAs

Annex V to the Environmental Protocol allows for the designation of Antarctic Specially Protected Areas (ASPAs) to protect values including areas of outstanding or representative terrestrial ecosystems. However, designated protected areas in the Antarctic Peninsula and Scotia Arc region are not immune to the impacts of fur seal damage to their terrestrial ecosystems. Of the 25 coastal ASPAs within the Antarctic Peninsula, South Shetland Islands and South Orkney Islands, and potentially within the enhanced summer dispersal range of fur seals, the management plans of 20 (80%) mention the seals, and five (20%) confirm them to have caused damage. Vegetation within ASPA No. 113 Litchfield Island, Arthur Harbour, Anvers Island, Palmer Archipelago, and ASPAs in the South Orkney Islands, has been particularly affected by fur seal impacts (Shears and Richard 1994). All three Antarctic Specially Managed Area (ASMA) management plans on the Antarctic Peninsula mention fur seals. That of ASMA No. 7 Southwest Anvers Island and Palmer Basin explicitly identified an increase in numbers in the past 20 years, noting that fur seals had ‘destroyed many sites of rich flora in the region’ (Antarctic Treaty Secretariat 2019).

### Practical management measures

Management activities to reduce seal impacts are potentially difficult and costly to put in place and generate conflict with regard to the protection of values associated with vegetation and terrestrial diversity compared to fur seal expansion. In essence, policymakers may have to establish whether it is more important to protect rare terrestrial communities (e.g. the unique communities developed on rare calcareous rocks on Signy Island) or fur seal populations that may have expanded following earlier human actions. However, it is the role of those environmental managers within national Antarctic programmes who must implement internationally agreed policy, to identify and put in place practical and affordable measures to provide the necessary protection. Annex II ‘Conservation of Antarctic fauna and flora’ to the Environmental Protocol states that ‘taking or harmful interference shall be prohibited, except in accordance with a permit’ (Article 3), with management of populations for conservation reasons not included in the list of reasons for provision of a permit.

**Fig. 5:**
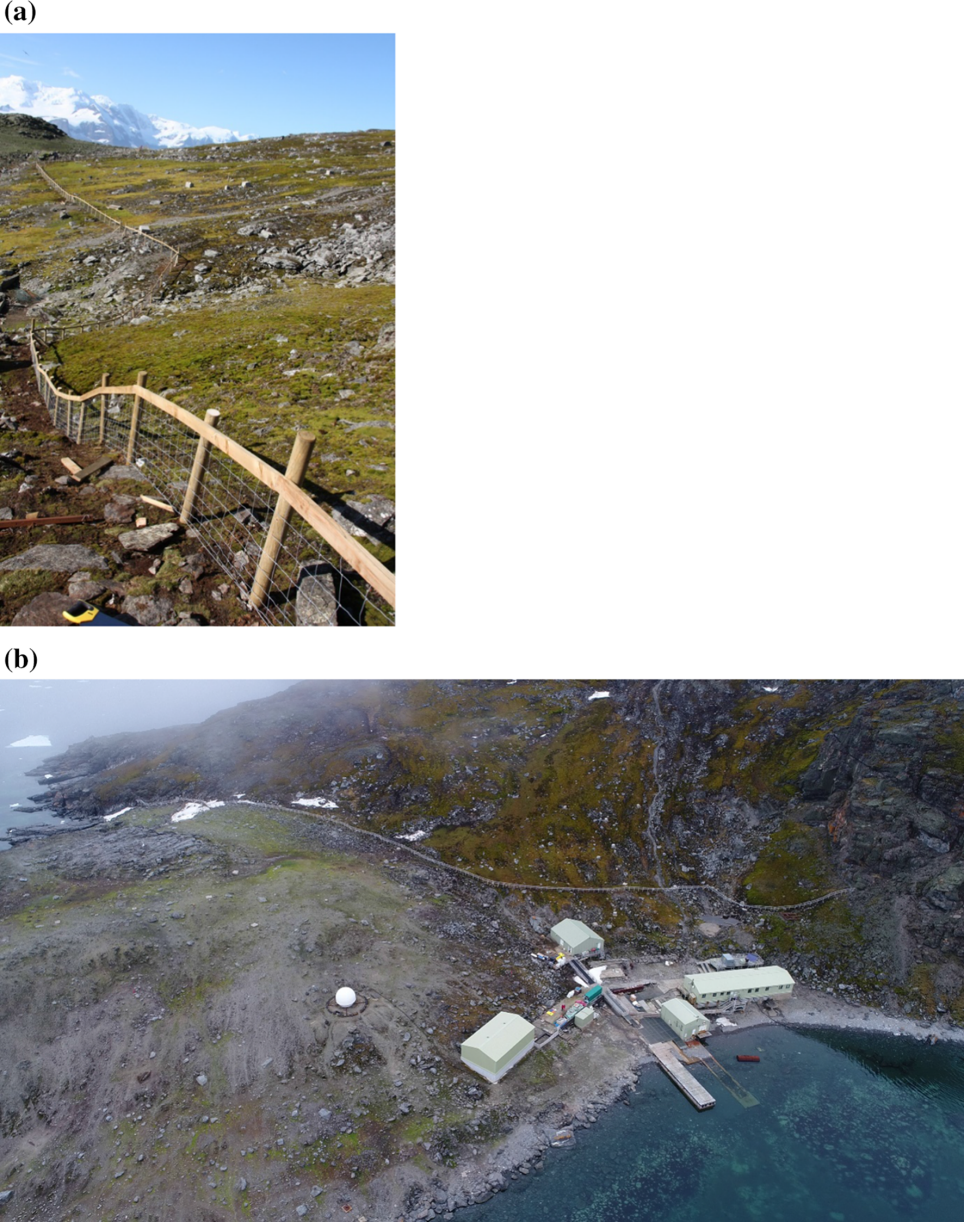
**a** The fence constructed on Signy Island to prevent fur seal access and protect one of the few remaining extensive areas of typical cryptogamic vegetation on the island (photo: M. Dunn). **b** Aerial view of the current Signy Station in Factory Cove, Signy Island, showing the position of the fence constructed across part of Berntsen Point to restrict Antarctic fur seal access to the richly vegetated ‘Backslope’ area leading up to Observation Bluff (see also Figs. 1, 3). While the research station area on Berntsen Point has been subject to human influence since the construction of a small whaling station in the early 1920s, the role of the fence in protecting the Backslope vegetation is clear (photo: A.P. Taylor and S. Adlard)

Earlier and current attempts to limit fur seal access to vegetated areas through the use of fencing have had mixed success, with most being abandoned and removed due to the need for on-going repair following persistent seal damage. The extent of damage to terrestrial ecosystems on Signy Island, and also to the neighbouring small Lynch Island (ASPA No. 110 close to the south coast of Coronation Island, declared primarily to protect its exceptional lawns of the native grass *Deschampsia antarctica*), led initially during the 1980s to attempts at active management through the installation of fences across seal access routes to parts of the islands. On Lynch Island, this was achieved by fencing off two narrow gullies which were the primary access route. However, this was only partially successful, with only very infrequent maintenance possible and seals finding other routes to access the lawns, and the approach was abandoned around the time that Signy research station became a summer-only operating station in the mid-1990s with much reduced logistical support capability. On Signy Island, similar small fences were used in the 1980s and 1990s to attempt to prevent access to some lakes (e.g. Tranquil Lake, see Fig. 3) that were important to lake monitoring programmes operating on the island at that time (e.g. see Pearce et al. 2005). The only area of terrestrial habitat currently subject to protection attempts is the ‘Backslope’ (unofficial name), from close to the Signy research station in Factory Cove, up to Observation Bluff, which includes a significant area of well-developed and representative maritime Antarctic vegetation. A fence was first constructed to restrict seal access to this area in the early 1990s, with only partial success. The fence was replaced and strengthened in the 2010/11 summer season and remains in place to the present day (Figs. 3, 5a, b), being largely effective.

The installation of means of physical protection of terrestrial habitats from what might be regarded as native biota raises important points of principle in the debate as to how future conservation might be achieved in the Antarctic, particularly relating to the need for prioritisation of different competing factors or values (seals vs. terrestrial ecosystems). This is not a subject addressed within the text of the Environmental Protocol or in discussions to date within the Committee for Environmental Protection. It is important to consider the multiple aspects of direct and indirect human intervention that provide the foundations of the current situation (see overview provided in Table 2). These range from the original uncontrolled exploitation of multiple marine resources and destabilisation of the Southern Ocean marine ecosystem, through the different aspects of anthropogenic climate change that have more recently facilitated fur seal expansion southwards, to the further pressure placed on availability of terrestrial habitat area by expansion of human facilities, research sites and visitor locations.

**Table 2:** Description of factors affecting level of fur seal trampling of coastal terrestrial habitats over the past c. 250 years

	Pre-seal exploitation era	During the peak seal exploitation era (late eighteenth and nineteenth centuries)	During seal population recovery period (early to late twentieth century)	1980s–2020s
Seal population levels	High numbers on South Georgia. Much lower, but variable, numbers (as estimated from seal hair presence in sediment cores) on Scotia Arc archipelagos	Seal populations almost driven to extinction on South Georgia and South Shetland Islands	From very low number, rapid population increase, particularly on South Georgia, to level similar to and potentially higher than during the pre-exploitation period	Large increase in numbers towards end of twentieth century, but with evidence of decline in the early twenty-first century potentially linked to complex combination of change in food availability and distribution, and possible competition with recovering whale populations
Seal distribution	Seal populations on sub-Antarctic islands, primarily South Georgia, with smaller populations on maritime Antarctic South Shetland Islands and South Sandwich Islands	Distribution dramatically decreased due to near extinction through over- exploitation	As populations recovered, breeding distribution recovered to pre-exploitation range	Increased southern distribution range of summer-dispersing male seals extending foraging and haul-out range, possibly facilitated by climate change driven retreat of sea ice extent and new foraging opportunities
Whale populations levels	Large whale populations in Southern Ocean, almost certainly larger than exist currently	Populations declined steeply due to overexploitation in the early and mid-twentieth century	Populations of some species slowly increased after whaling industry collapse in the 1960s and international moratorium on whaling introduced in 1986	Increasing recovery of some whale populations in the Southern Ocean
Food availability for fur seals (e.g. krill)	Moderate levels of food availability, as krill also supported some whale, penguin and fish populations	High levels of krill availability due to decline in seal populations and later decline in whale populations, both through human exploitation	Continued high krill availability with slow recovery of whale populations, facilitating fur seal population explosion	Krill availability may be declining as whale populations recover; also potential impact of human krill fishery
Levels of damage to terrestrial communities	Terrestrial ecosystems strongly impacted close to pre-exploitation breeding concentrations of seals (primarily South Georgia, possibly parts of South Shetland Islands)	Little or no damage to terrestrial communities within previous breeding range due to general absence of fur seals and also reduced numbers of elephant seals	Rapid return of damage to coastal sub-Antarctic (South Georgia) terrestrial communities as breeding population of seals recovered; new damage towards the end of the twentieth century to areas previously not exposed to seals (primarily South Orkney Islands) due to high numbers of summer-dispersing males hauling out at coastal sites	Further high levels of damage extending southwards along western Antarctic Peninsula as new coastline and haul-out sites become available due to sea ice retreat linked with climate change

## Conclusions

With predicted warming trends continuing throughout the twenty-first century, as well as possible further expansion of summer-dispersing male seals to more southerly latitudes (e.g. to Pine Island Bay, 75° S, 102° W, or farther) where accessible ice-free terrain certainly exists supporting Adélie penguin colonies, consideration should be given to the potential for the fur seal breeding range to expand to suitable sites along the Antarctic Peninsula. The fur seal expansion, and its associated impacts, is clearly not simply a process driven by natural causes occurring within the native range of this seal species, and rather has a complex combination of very strong and originally anthropogenic drivers (Table 2). Furthermore, with increasing emphasis on the adoption of effective conservation and environmental protection practices since the Environmental Protocol entered into force in 1998, there is considerable effort and pressure to reduce and control sources of direct human impact such as trampling and vehicle damage in the Antarctic terrestrial environment. Given the strongly contrasting relative extent and scale of impacts of such human activities and the newly occupied fur seal range, the relative importance of contemporary direct human impacts on local environments and of those impacts—both direct and indirect—arising as consequences of previous interactions, will require further consideration by policymakers.

Examples, such as the foregoing, highlight the importance of considering all relevant factors when assessing the impacts of both distant and local human influences on Antarctic terrestrial and marine ecosystems. They also expose the tensions in decision-making regarding actions required to control and mitigate these impacts in order to provide effective protection to these ecosystems and their contained biodiversity and communities. It is clear that many different factors, both of human origin and of the natural environment, may act in synergy and disentangling their subtleties is often more challenging and complex than previously realised.
